# Trajectories of physical activity and sedentary time in Norwegian children aged 3–9 years: a 5-year longitudinal study

**DOI:** 10.1186/s12966-022-01286-0

**Published:** 2022-06-11

**Authors:** Eivind Aadland, Anthony D. Okely, Ada Kristine Ofrim Nilsen

**Affiliations:** 1 Faculty of Education, Arts and Sports, Department of Sport, Food and Natural Sciences, Western Norway University of Applied Sciences, Campus Sogndal, Sogndal, Norway; 2 University of Wollongong, Early Start and School of Health and Society, Wollongong, Australia

**Keywords:** Children, Accelerometer, Longitudinal, Preschool, School

## Abstract

**Background:**

Limited evidence exists regarding the longitudinal development of physical activity during early to mid childhood. The aim of this study was to determine physical activity and sedentary time trajectories in children aged 3‒9 years from Western Norway.

**Methods:**

A sample of 294 children (51% boys; aged 3‒5 years at baseline) from the Sogn og Fjordane Preschool Physical Activity Study was followed annually over 5 years (2015‒2019). Physical activity was measured every autumn during this period using hip-based accelerometry (ActiGraph GT3X+). Data was processed as counts. We used linear mixed models to analyse the data. Primary analyses included trajectories for total and intensity-specific physical activity (light, moderate, vigorous, and moderate to vigorous intensity) and sedentary time for boys and girls using 1-s epoch. Secondary analyses included trajectories for weekdays versus weekend days, preschool/school hours versus after school hours, and 1- versus 60-s epoch lengths.

**Results:**

Over the total day, significant associations with age were found for boys and girls for all physical activity intensities and sedentary time (*p* < .001). Total physical activity peaked at age 5, whereas intensity-specific physical activity levels peaked at age 3 to 8 years (light intensity: 3–4 years; moderate intensity: 4–7 years; vigorous intensity: 7–8 years; moderate to vigorous intensity: 5–8 years). Boys had higher physical activity levels and more favourable trajectories than girls. Sedentary time increased from ages 3 to 9. Changes over time were larger for weekdays than for weekend days and larger for preschool/school hours than for after school hours. The use of a 60-s epoch resulted in larger changes over time than a 1-s epoch.

**Conclusions:**

Our findings suggest physical activity levels peaked between the ages of 3 and 8 years. Finding ways to prevent decline in physical activity during the transition from preschool to primary school is important, especially for girls. Standardising epoch length will help when comparing physical activity and sedentary behaviour across studies.

**Supplementary Information:**

The online version contains supplementary material available at 10.1186/s12966-022-01286-0.

## Background

Physical activity (PA) promotes physical development and metabolic health in children and youth [1, 2]. There is also increasing evidence that PA beneficially affects brain health, cognition, and learning outcomes [3, 4]. Thus, sufficient levels of PA should be attained throughout childhood to ensure optimal health and development. There are growing concerns regarding low PA levels among children and youth [5, 6] and PA trajectories appear to be declining from a relatively early age [5, 7]. Although evidence consistently shows that PA levels decline during adolescence [5, 7], discrepancies exist in the literature with respect to how PA levels change during early (ages 3 to 5 years) to mid (ages 6 to 9) childhood [5, 7–20].

Findings from the International Children’s Accelerometry Database (ICAD) suggest children exhibit the highest levels of total PA and moderate- to vigorous-intensity PA (MVPA), and lowest sedentary time (SED), at 5–6 years. After this age, PA gradually declines and SED increases with age [5]. Although the harmonized data reduction approach in the ICAD is a strength of the study, the findings are limited by being mostly derived from cross-sectional studies. A peak MVPA level at ages 5–6 is partly supported by a recent meta-analysis of longitudinal studies [7], although this study suggested that the peak age differed by sex; the peak MVPA was achieved at age 6 for girls, whereas boys achieved their peak at age 8. Studies with long-term follow-up and multiple measurement timepoints suggest that the peak PA level is achieved at age 3 (assessed from age 3 to 7 years) [8], 5 (assessed from age 1 to 5) [9], 7 (assessed from age 7 to 15 years) [10], 8 (assessed from age 6 to 11) [11], 9 (assessed from age 5 to 15 years) [12], and 5–11 years (assessed from age 5 to 15 years) [13]. In the latter study, levels of MVPA peaked prior to vigorous-intensity PA (VPA), and PA levels among girls peaked prior to boys [13]. The peak levels of VPA at ages 8 and 11 for girls and boys, respectively, are in contrast to the decline in VPA from age 5 (6.9% per year) found in the ICAD [12] and from age 7 found in the longitudinal Gateshead Millennium study [15]. Moreover, the meta-analysis by Farooq [7] suggests the decline in MVPA with increased age was greater in girls than in boys and greater on weekends than on weekdays from age 3 to 18 years. Thus, there is considerable uncertainty with respect to PA trajectories during early childhood and to what extent they differ by sex and PA intensity, as well as across weekdays and weekend days.

There is a lack of longitudinal studies of PA in preschool-aged children [7] and few studies capture the transition from preschool to school. Additionally, the few existing studies show conflicting findings with respect to the development of PA in this period. The longitudinal study by Taylor et al. [8] found a decline in New Zealand children’s PA from age 3 to the start of primary school and an increasing trend from the start of primary school to the age of 7 (a U-shaped curve). This finding differs from other studies in this age group, which have shown increasing PA levels during the preschool period (up to an age of 6 years) [16–18] and declining PA levels after starting school [5, 7]. Discrepancies also exist between Norwegian studies, where cross-sectional surveillance data suggest PA is reduced in schoolchildren from age 6 to 9 [19], whereas a 2-year longitudinal study following children from preschool to school showed increasing PA levels from age 3 to 8 [20].

The meta-analysis by Farooq et al. [7] did not apply a harmonized approach to accelerometry, hence, findings might be influenced by the use of, among other factors, different PA intensity cut points and epochs [21, 22], in addition to inherent variation across countries. As children’s PA patterns are sporadic and characterized by short intermittent bursts of activity, a shorter epoch is needed to capture the higher and lower extremes of the intensity spectrum correctly [23, 24]. The ICAD study is limited by a long epoch (60-s) [5, 14] and the meta-analysis by Farooq et al. [7] by varying epochs. These inconsistencies in data reduction approaches will obfuscate the findings. Finally, since most studies summarize moderate and vigorous intensities (i.e., MVPA), less evidence is available for trajectories of VPA, which is the intensity most strongly associated with multiple health and developmental outcomes in children, including physical fitness, body composition, cardiometabolic health, and motor skills [1, 23, 25].

The primary aim of this study was to describe the longitudinal changes in PA and SED over a 5-year period among Norwegian children aged 3 to 9 years, including potential differences between boys and girls, capturing the transition from preschool to primary school. Secondary aims were to investigate how changes over time varied by type of day (weekdays versus weekend days) and time of day (preschool/school hours versus after school hours) and how changes varied by epoch length (1- versus 60-s).

## Methods

This was a longitudinal analysis based on data from the *Sogn og Fjordane Preschool Physical Activity Study (PRESPAS) follow-up study* conducted 2015–2019. PRESPAS was a cross-sectional study conducted in the county of Sogn og Fjordane, a rural area in Western Norway, between September 2015 and June 2016 and involved a total of 1308 children aged 2.7–6.5 years (born in 2010–2012) from 68 preschools (response rate 68%) from 14 municipalities [16]. The present study (PRESPAS follow-up) was based on a convenience subsample of 376 invited children from 20 preschools (all preschools from 3 municipalities) who provided data at baseline and at one or more timepoints during a 5-year follow-up period. The PRESPAS follow-up study was designed to capture the transition from preschool to primary school. During this period, children’s PA levels were assessed annually during September–October.

Parents of all participating children received oral and written information about the study and provided written consent prior to testing. Preschools and primary schools received information and agreed to participate in the study. We explained the procedures according to the children’s level of understanding. The Norwegian Centre for Research Data (NSD) approved the study (reference numbers: 39061 and 48016).

### Procedures

#### Physical activity measurement

PA was measured using the ActiGraph GT3X+ accelerometer (ActiGraph, LLC, Pensacola, Florida, USA) [26]. Children wore an elastic belt with the accelerometer on the right hip and were instructed to wear the monitor at all times except during water-based activities and while sleeping (at night) for 14 consecutive days. Accelerometers were initialized at a sampling rate of 30 Hz. Files were analysed restricted to hours 06:00 to 23:59 for a total day (weekdays and weekend days), 08:30 to 15:29 for preschool/school hours (weekdays only), and 15:30 to 23:59 for after school hours (weekdays only). Count-based data from the vertical axis were analysed using a 1-s epoch to capture low and high intensity PA [23]. We also included a sensitivity analysis using a 60-s epoch to determine the influence of epoch length on PA trajectories. Periods of ≥ 20 min of zero counts were defined as non-wear time [27]. We applied wear time requirements of ≥ 480 min/day and ≥ 3 weekdays and ≥ 1 weekend day for the total day [28, 29]. The criteria were ≥ 3 weekdays of valid preschool/school hours (≥ 270 min/day), and ≥ 3 weekdays of valid after school hours (≥ 180 min/day). Outcomes were total PA (counts per minute [cpm]), SED (≤ 100 cpm), and intensity-specific PA defined as light-intensity PA (LPA) (101–2295 cpm), moderate-intensity PA (MPA) (2296–4011), VPA (≥ 4012 cpm), and MVPA (≥ 2296 cpm) (min/day), as proposed by Evenson et al. [30, 31]. The KineSoft analytical software version 3.3.80 (KineSoft, Loughborough, UK) was used for all analyses.

#### Anthropometrics and demographics

We assessed children’s body mass and height during preschool hours. Body mass was measured to the nearest 0.1 kg using an electronic scale (Seca 899, SECA GmbH, Hamburg, Germany) and height was measured to the nearest 0.1 cm with a portable stadiometer (Seca 217, SECA GmbH, Hamburg, Germany). Body mass index (BMI, kg/m^2^) was calculated, and children were classified as normal weight (including underweight), overweight, or obese based on the criteria proposed by Cole et al. [32]. Parental education (highest education level of mother or father) was assessed using a questionnaire completed by each child’s mother and/or father every autumn. Place of birth (Norway or other) were assessed for children and parents at baseline.

#### Statistical analysis

Results were reported as frequencies, means and standard deviations (SD). PA trajectories were analysed using a linear mixed model including random intercept for children. This model allowed for including all observations from all children, irrespective of the number of measurements, in the analyses. A linear mixed model is a powerful approach for analysing longitudinal data, allowing for determination of unbiased estimates in case of missing data [33, 34] or in case of severe violations of its assumptions [35]. We modelled age as a categorical variable from ages 3 to 9 (children aged 2.5–3.4 years were defined as 3 years etc.) to account for non-linear changes over time and verified that residuals were normally distributed for all models. In the primary analyses, we determined the main effect of age for the whole sample while controlling for sex and tested the moderating effect of sex on PA trajectories by adding the interaction term age*sex to this model. In the secondary analyses, we extended this model to examine whether PA trajectories differed across type of day (weekdays versus weekend days) or time of day (preschool/school hours versus after school hours on weekdays) by adding age*type of day or age*time of day interactions. We also tested the age*epoch interaction to examine whether PA trajectories for the total day differed for 1- or 60-s epochs. As wear time is expected to increase with age due to reduced sleep duration [36], we did not control for accelerometer wear time in the analyses. PA trajectories were described, but differences between specific age groups (e.g., 5 versus 6 years) were not formally tested because we did not have specific a priori hypotheses about the trajectories. Stability (tracking) of PA over time was determined for 1-s epoch data using intraclass correlations (ICCs) derived from the linear mixed model (i.e., determining agreement based on a consistency definition [37]). The sample included in the follow-up study was compared to the larger PRESPAS sample using linear mixed models (continuous outcomes) or generalized estimating equations (categorical outcomes) taking into account clustering within preschools. Since the follow-up sample was assessed during the autumn 2015 and the larger PRESPAS sample during 2015–2016, the follow-up sample was younger than the larger sample. Height, body mass, and BMI were therefore adjusted for age in these analyses. PA and SED were not compared between samples as potential differences cannot be distinguished from seasonal effects [16]. All analyses were performed using IBM SPSS v. 27 (IBM SPSS Statistics for Windows, Armonk, NY; IBM Corp., USA). *P*-values < 0.05 indicated statistically significant findings for main effects, whereas *p*-values < 0.10 indicated statistically significant interactions.

## Results

We included 294 (78%) children who provided valid data for all analysed contexts (≥ 3 weekdays, ≥ 1 weekend day, ≥ 3 weekday preschool/school hours, and ≥ 3 weekday after school hours) for at least 2 timepoints (Table 1 and Supplemental Fig. 1, Additional file 1). 19 (6%), 32 (11%), 77 (26%), and 166 (56%) children provided valid data for 2, 3, 4, and 5 timepoints, respectively, summing to a total of 1272 unique observations (87% of potential observations) for each of the contexts. The included sample did not differ from the larger PRESPAS sample with respect to height, body mass, BMI, proportion of boys and girls, or proportion classified as normal weight, overweight, or obese (p ≥ 0.566). However, the follow-up sample had a higher parental education level (*p* = 0.007) and a higher proportion of children and parents born in Norway (*p* = 0.011–0.028) (Table 1).

**Table 1:** Characteristics of the children included in the PRESPAS study and the PRESPAS follow-up study

	**PRESPAS**	**PRESPAS follow-up**				
	**2015–2016**	**2015**	**2016**	**2017**	**2018**	**2019**
n	1308	253	282	285	282	282
Age (years)	4.7 (0.9)	4.3 (0.9)	5.3 (0.9)	6.3 (0.9)	7.3 (0.9)	8.3 (0.9)
Boys (%)	52	51	51	51	51	51
Body mass (kg)	19.3 (3.2)	18.5 (3.0)	20.9 (3.6)	23.4 (4.3)	26.8 (5.2)	30.4 (6.3)
Height (cm)	109 (7)	106 (8)	114 (8)	120 (8)	126 (7)	133 (7)
Body mass index (kg/m^2^)	16.2 (1.4)	16.3 (1.3)	16.1 (1.4)	16.0 (1.6)	16.5 (2.0)	16.9 (2.3)
Overweight/Obese (%)						
Under or normal weight	81.9	81.8	83.4	84.7	83.6	83.9
Overweight	15.7	17.4	15.1	12.3	12.0	12.1
Obese	2.4	0.8	1.5	3.0	4.4	4.0
Parental education level (%)						
Upper secondary school	22.8	13.4	12.0	11.7	10.8	10.7
University < 4 years	25.9	28.7	27.4	27.4	27.3	28.9
University ≥ 4 years	51.2	57.9	60.6	60.9	61.9	60.4
Born in Norway (%)						
Child	96.6	99.6	98.7	99.1	99.2	98.7
Mother/father	87.1/86.8	92.4/91.6	92.0/91.2	92.3/91.9	94.1/93.2	93.4/93.0

For the total PRESPAS sample *n* = 1087–1140 for child origin and parental origin and education, and *n* = 1249 for body mass, height, and BMI. For the PRESPAS follow-up sample *n* = 225–249 for child origin and parental origin and education, and *n* = 241–276 for body mass, height, and BMI

**Fig. 1:**
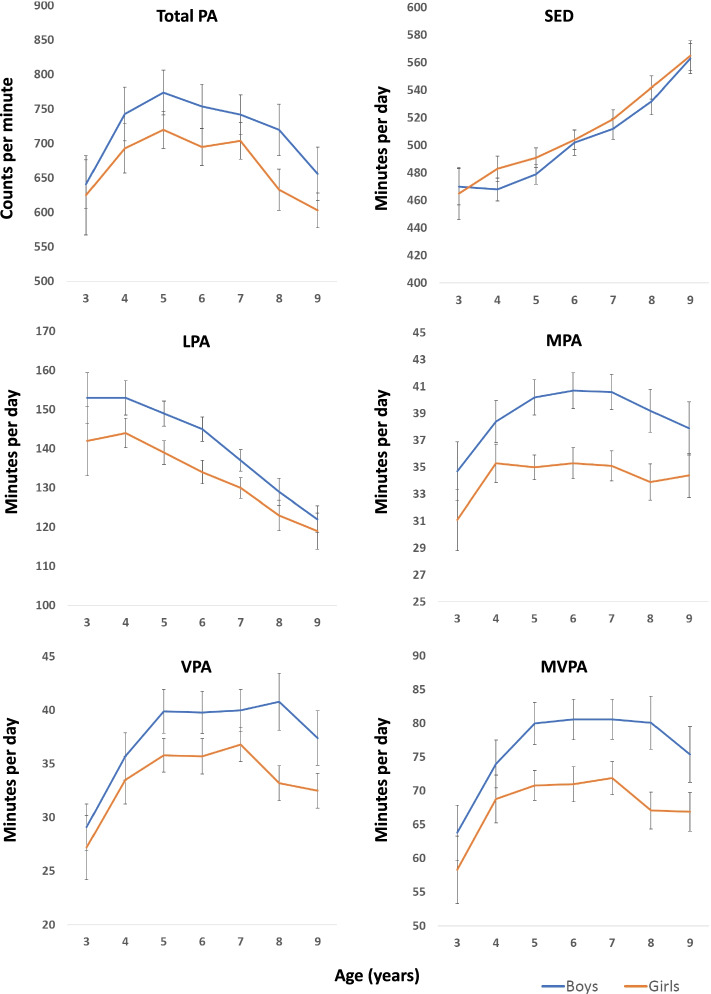
Trajectories for physical activity and sedentary time in boys and girls aged 3–9 years. Graphs show means and 95% confidence intervals. Epoch length: 1-s. *PA* physical activity, *SED* sedentary time, *LPA *light physical activity, *MPA* moderate physical activity, *VPA* vigorous physical activity, *MVPA* moderate- to vigorous physical activity

### Physical activity trajectories for the total day

Development of PA and SED (minutes/day) over the total day from ages 3 to 9 is shown in Fig. 1 and Table 2 (proportions are shown in Supplemental Fig. 2 and Supplemental Table 1, Additional file 1). While trajectories were similar for boys and girls for total PA (p for age*sex = 0.542), SED (*p* = 0.612), and MVPA (*p* = 0.124), differing trajectories were indicated for LPA (*p* = 0.066), MPA (*p* = 0.098), VPA (*p* = 0.065). There was no age*sex interaction for wear time (*p* = 0.224). Trajectories with age were significant for boys and girls for all variables (*p* < 0.001). For boys and girls, total PA increased from ages 3 to 5 and thereafter declined, whereas SED increased from ages 3 to 9. LPA declined from ages 4 to 9, with boys having a higher initial level and a greater decline than girls. Generally, MPA, VPA, and MVPA increased from age 3 to 5, thereafter plateaued, and then declined from ages 7 to 9. Boys had a greater increase and a later decline than girls for minutes per day spent in moderate- and vigorous intensities.

**Table 2:** Mean (SD) PA levels for the total day across age and sex

	**Age**						
	3	4	5	6	7	8	9
	**Boys**						
Number of observations							
Potential n	38	83	136	146	155	110	87
Included n (%)	30 (79)	67 (81)	121 (89)	126 (86)	134 (86)	96 (87)	76 (87)
Wear days (n)	12.9 (1.5)	12.7 (1.8)	12.3 (2.1)	12.6 (3.0)	12.4 (2.8)	12.1 (2.6)	12.4 (2.1)
Wear time (min/day)	688 (39)	695 (32)	709 (38)	727 (51)	729 (45)	741 (41)	761 (41)
Total PA (cpm)	641 (99)	743 (162)	774 (182)	754 (182)	742 (167)	720 (188)	656 (172)
SED (min/day)	470 (37)	468 (35)	479 (41)	502 (53)	512 (45)	532 (49)	563 (49)
LPA (min/day)	153 (18)	152 (18)	149 (18)	145 (18)	137 (16)	129 (17)	122 (15)
MPA (min/day)	35 (6)	38 (7)	40 (7)	41 (8)	41 (8)	39 (8)	38 (9)
VPA (min/day)	29 (6)	36 (9)	40 (11)	40 (11)	40 (11)	41 (13)	37 (11)
MVPA (min/day)	64 (11)	74 (15)	80 (18)	81 (17)	81 (18)	80 (20)	75 (19)
	**Girls**						
Number of observations							
Potential n	34	81	129	138	148	108	77
Included n (%)	23 (68)	68 (84)	114 (88)	127 (92)	131 (89)	95 (88)	64 (83)
Wear days (n)	12.7 (1.7)	12.8 (1.9)	12.6 (2.2)	12.3 (2.6)	12.6 (3.0)	11.8 (2.4)	12.0 (2.3)
Wear time (min/day)	665 (49)	696 (39)	701 (36)	708 (38)	721 (38)	732 (40)	751 (50)
Total PA (cpm)	625 (141)	693 (150)	720 (146)	695 (153)	704 (156)	633 (150)	603 (104)
SED (min/day)	465 (47)	483 (39)	491 (39)	504 (40)	519 (40)	542 (42)	565 (44)
LPA (min/day)	142 (22)	144 (16)	139 (16)	134 (17)	130 (15)	123 (19)	119 (19)
MPA (min/day)	31 (6)	35 (6)	35 (5)	35 (7)	35 (6)	34 (7)	34(7)
VPA (min/day)	27 (7)	34 (9)	36 (8)	36 (9)	37 (9)	33 (8)	32 (7)
MVPA (min/day)	58 (12)	69 (15)	71 (12)	71 (15)	72 (14)	67 (14)	67 (12)

*PA* Physical activity, *SED* Sedentary time, *LPA* Light physical activity, *MPA* Moderate physical activity, *VPA* Vigorous physical activity, *MVPA* Moderate- to vigorous physical activity

**Fig. 2:**
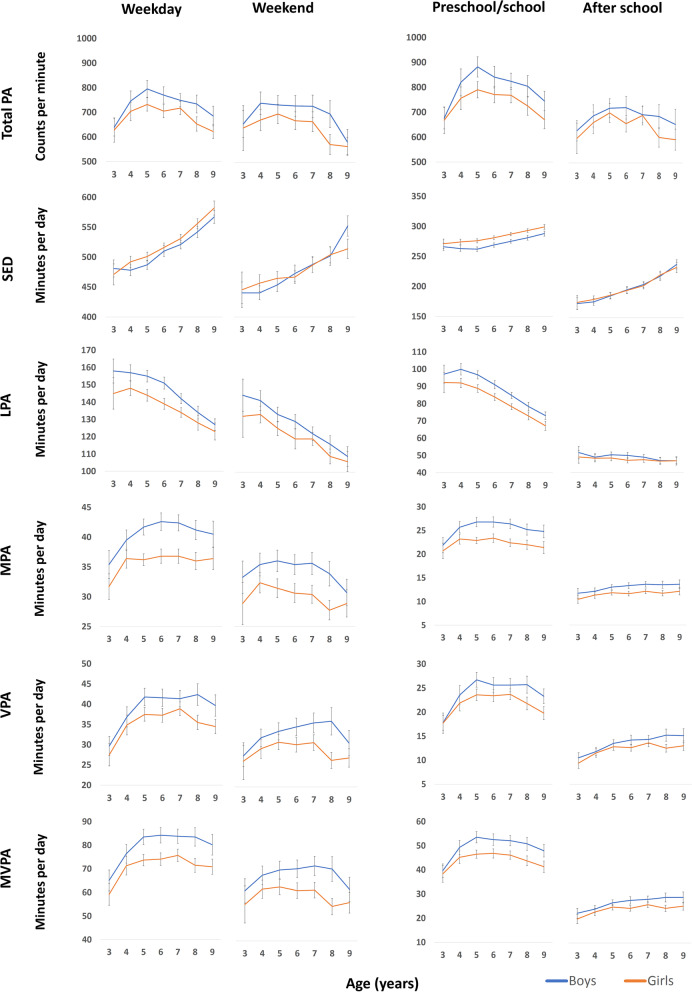
Trajectories for physical activity and sedentary time across type of day and time of day. Graphs show means and 95% confidence intervals. Weekdays and weekend days (left); preschool/school hours and after school hours (right). Epoch length: 1-s. *PA* physical activity, *SED* sedentary time, *LPA* light physical activity, *MPA* moderate physical activity, *VPA* vigorous physical activity, *MVPA* moderate- to vigorous physical activity

### Physical activity trajectories across type of day and time of day

Figure 2 and Supplemental Tables 2–5 (Additional file 1) show the PA trajectories for weekdays versus weekend days and for preschool/school hours versus after school hours from age 3 to 9. Different trajectories were found for weekdays versus weekend days for total PA, MPA, VPA, and MVPA (p for age*type of day =  < 0.001–0.072), and for all variables except for total PA for preschool/school versus after school hours (p for age*time of day < 0.001). The different trajectories for weekdays and weekend days reflected a steeper and greater increase with age for total PA, MPA, VPA, and MVPA for weekdays than for weekend days. The different trajectories for SED and all PA intensities for preschool/school hours versus afterschool hours reflected a steeper increase (or a less steep decrease) with age for preschool/school hours than for after school hours. While MPA, VPA, and MVPA increased from ages 3 to ages 4–5 during preschool/school hours and then plateaued before declined, these variables showed a rather stable increase from ages 3 to 9 during after school hours. Note that the wear time was constant over time during preschool/school hours (402 (boys)/401 (girls) and 409 (boys)/407 (girls) min/day for children aged 3 and 9 years, respectively), whereas it increased with age during the after school hours (248 (boys)/242 (girls) and 315 (boys)/308 (girls) min/day for children aged 3 and 9 years, respectively) (Supplemental Tables 4–5, Additional file 1).

### Comparison of physical activity trajectories using 1- and 60-s epoch lengths

**Fig. 3:**
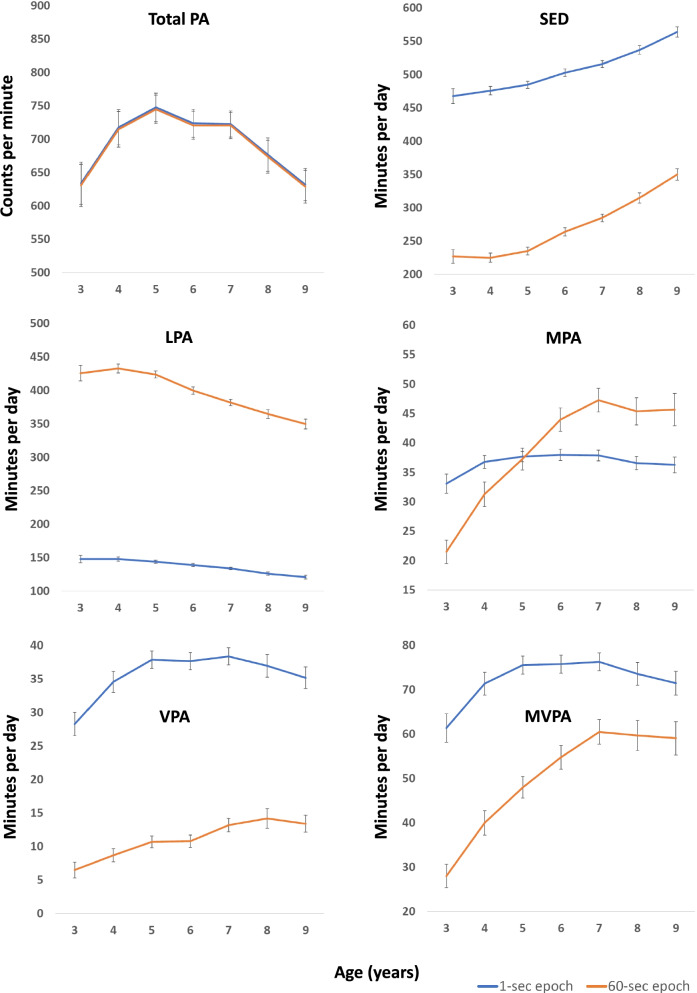
Trajectories for physical activity and sedentary time using 1- versus 60-s epoch. Graphs show means and 95% confidence intervals. *PA* physical activity, *SED* sedentary time, *LPA* light physical activity, *MPA* moderate physical activity, *VPA* vigorous physical activity, *MVPA* moderate- to vigorous physical activity

Figure 3 and Supplemental Table 6 (Additional file 1) shows the trajectories for a 1- or 60-s epoch (Supplemental Fig. 3 and Supplemental Table 7 (Additional file 1) show the trajectories for boys and girls using a 60-s epoch). There were large differences in PA levels between the epoch lengths for all variables, except for total PA and MPA. A 1-s epoch resulted in higher SED (503 versus 264 min/day for a 6-year-old), VPA (38 versus 11 min/day for a 6-year-old), and MVPA (76 versus 55 min/day for a 6-year-old), and a lower LPA (139 versus 400 min/day for a 6-year-old) than a 60-s epoch. Different trajectories were found for 1- versus 60-s epoch for all PA intensities (p for age*epoch =  < 0.001), while trajectories were similar for total PA (*p* = 1.000). Compared to a 1-s epoch, a 60-s epoch resulted in a steeper increase with age for SED (123 versus 96 min/day increase from ages 3 to 9), MPA (24 versus 3 min/day increase from ages 3 to 9), and MVPA (31 versus 10 min/day increase from ages 3 to 9), and a steeper decline for LPA (76 versus 27 min/day decrease from ages 3 to 9). For VPA, there was a relatively stable increase with age for a 60-s epoch, whereas there was an increase, plateau, and then a decrease with age for a 1-s epoch, resulting in similar changes from ages 3 to 9 (6 versus 7 min/day, respectively).

### Stability of physical activity trajectories

Table 3 shows the stability of PA and SED for the total day and across type of day and time of day over the 5-year period. For the total day, ICCs were 0.47–0.59 for boys and 0.50–0.54 for girls. The ICCs were lowest for the weekend (0.26–0.41 for boys and 0.30–0.38 for girls) but comparable for weekdays, preschool/school hours, and after school hours (0.38–0.58 for boys and 0.38–0.57 for girls).

**Table 3:** Stability (ICC) of PA over the 5-year period

	**Total day**	**Weekday**	**Weekend**	**Preschool/school**	**After school**
**Boys**					
Total PA (cpm)	0.58	0.52	0.38	0.42	0.45
SED (min/day)	0.48	0.48	0.26	0.42	0.58
LPA (min/day)	0.47	0.42	0.36	0.38	0.45
MPA (min/day)	0.49	0.45	0.32	0.40	0.43
VPA (min/day)	0.59	0.55	0.41	0.47	0.49
MVPA (min/day)	0.57	0.53	0.38	0.47	0.48
**Girls**					
Total PA (cpm)	0.50	0.48	0.30	0.39	0.38
SED (min/day)	0.53	0.50	0.30	0.50	0.57
LPA (min/day)	0.54	0.53	0.38	0.52	0.47
MPA (min/day)	0.54	0.53	0.33	0.50	0.41
VPA (min/day)	0.51	0.48	0.33	0.42	0.40
MVPA (min/day)	0.54	0.52	0.33	0.49	0.41

*PA* Physical activity, *SED* sedentary time, *LPA* Light physical activity, *MPA* Moderate physical activity, *VPA* Vigorous physical activity, *MVPA* Moderate- to vigorous physical activity

## Discussion

In this longitudinal study, trajectories of total and intensity-specific PA and SED were examined in Norwegian children aged 3 to 9 years. Boys generally had more favourable PA levels and trajectories than girls, including a steeper increase and a later plateau and decline in PA of moderate and vigorous intensities. Trajectories were generally similar across weekdays and weekend days but differed for preschool/school hours and after school hours. Epoch length had a major influence on levels and trajectories for all PA intensities and SED. Being one of a small number of studies to follow a relatively large sample of young children from the preschool to the school setting, these findings provide further understanding of how PA develops with age across the transition from preschool to school, where prior studies suggest the well-known decline in PA during childhood begins.

While evidence generally support an increase in SED from the age of 3 onwards [5, 11, 12, 16, 20], the evidence on trajectories of PA across childhood is equivocal [5, 7, 8, 10, 12–14, 17, 18, 25, 34]. Part of this uncertainty results from the use of different accelerometer data reduction methodologies across studies [7, 21, 23]. Most studies show an increase in PA with age during preschool years (3–4/5 years of age) [5, 9, 16–18] and a decline during school years (from age 5/6) [5, 7, 12–15, 19]. The evidence on the timing of the peak PA level as determined by longitudinal studies is, however, conflicting and varies from 3 to 11 years depending on sex and intensity [7–13]. Few studies have followed large samples of young children over several years capturing the transition from preschool to school. One study that captured trajectories with multiple measurements across this transition, showed a U-shaped development from ages 3 to 7 among 242 children from New Zealand [8], which is contrary to trajectories described by the present study and the prevailing longitudinal evidence [7, 9, 10, 12, 13]. Our findings are consistent with most previous studies [7, 10, 12, 13], showing that PA levels (total day) peak at 3 to 8 years depending on sex, intensity, and, to some extent, epoch length. While LPA peaks at age 3–4 and total PA level (cpm) peaks at age 5 for both boys and girls, both MPA, VPA, and MVPA appear to peak at age 7 in girls and age 8 in boys in the present study. Interestingly, the peaks for moderate and vigorous intensities are considerably more pronounced for a 60-s epoch, for which we found clear increases in PA levels from the age of 3, than for a 1-s epoch, for which we found minor changes after age 4 in girls and age 5 in boys. SED increases from age 3 in girls and age 4 in boys. Later peaks for boys than for girls and for VPA than for MPA support previous longitudinal evidence [7, 13].

Our findings show smaller declines with age than Norwegian surveillance studies [19] and international data from the ICAD [5, 14]. Based on representative samples of 4406 Norwegian 6- and 9-year-olds in 2011 and 2018, Steene-Johannessen [19] showed significant declines for both total PA (100–132 cpm) and MVPA (8–12 min/day) between children aged 6 and 9 using a 10-s epoch. For comparison, we found smaller declines for total PA (< 94 cpm), while MVPA declined by 5 min/day using a 1-s epoch and increased by 3–6 min/day using a 60-s epoch. These findings further contrast findings from the ICAD, showing declined MVPA [5] and VPA [14] from the age of 5–6 years based on a 60-s epoch, though MVPA declined less than 1% until age 9–10. We have no clear explanation for the apparent discrepancies across studies and study designs beyond inherent variation among populations, possible methodological differences in handling accelerometry data, and possible secular trends influencing the comparison of our data collected 2015–2019 with older ICAD data.

While ICAD data suggest the transition from the preschool to the more formal school setting had a negative influence on PA trajectories, our findings, consistent with other longitudinal studies [10, 12, 13], suggest that PA of moderate and vigorous intensities do not decline when enrolled in school. However, previous longitudinal studies have not provided analyses for separate settings (i.e., preschool/school hours versus after school hours) across this transition. Our findings show that preschools and schools are important environments for children’s movement opportunities, given the higher PA levels during weekdays than weekend days and during preschool/school hours than the after school hours on weekdays, as also shown previously for the larger PRESPAS sample [38]. We found that MVPA increased by 15 min/day in boys and 10 min/day in girls on weekdays, compared to 2 min/day in boys and 0 min/day in girls on weekend days from age 3 to 9, reinforcing that children have the most positive trajectories over this age span on weekdays. This finding is consistent with the findings by the meta-analysis by Farooq et al. [7] that show a more pronounced decline in MVPA with age during weekends than weekdays. A closer look at trajectories during preschool/school hours showed that being introduced to formal schooling seems to affect PA levels negatively. We observed earlier peaks in MVPA (age 5 in both boys and girls) during preschool/school hours than for the total day (age 7–8). MVPA decreased by 4–5 min/day during school time but increased by 1 min/day during after school hours from ages 6 to 9. The positive trajectories during after school hours compared to preschool/school hours is, however, explained by the increased wear time of 66–67 min/day during after school hours, contrasting the stable (and restricted) wear time during preschool/school hours. Thus, when expressed as the proportion of wear time, MVPA levels are stable in both boys (8.9–9.2%) and girls (8.3–8.1%) from ages 3 to 9 during after school hours. Given that decreased sleep time [36] and thus increased opportunities for movement is a natural part of a child’s development, we did not adjust for wear time in our analyses.

Consistent with previous studies [5, 7, 16, 19, 37], we found that boys had more positive PA trajectories than girls from a young age, had a later peak for moderate- and vigorous intensities, and were more active throughout the follow-up period. Of particular interest, boys appeared to have more pronounced positive MVPA trajectories than girls during preschool/school hours (increase of 14 min/day for boys versus 8 min/day for girls from ages 3 to 5 and 8 min/day for boys versus 3 min/day for girls from ages 3 to 9, respectively) compared with after school hours (increase of 5 min/day for boys versus 4 min/day for girls from age 3 to 5 and 7 min/day for boys versus 5 min/day for girls from age 3 to 9, respectively). These findings support those by Nilsen et al. [38], showing that boys, older children, and highly active children benefit most from the preschool environment with regards to MVPA. This pattern likely results from different play preferences at preschool [38, 39], but also suggests the preschool movement environment and pedagogical approach may suit boys better than girls and that initiatives specifically should address girls’ movement needs and preferences. Thus, preschool interventions should be designed to specifically promote PA in girls by designing PA programs meeting girls’ preferences and by raising the awareness of these gendered activity patterns by preschool staff through professional development. Such early initiatives could hopefully have immediate and long-lasting effects on PA levels in girls throughout childhood.

It is well known that the epoch length is fundamental for the capture of intensity-specific PA using accelerometry in children due to children’s sporadic and intermittent bursts of activity [23, 24, 40]. Summation of PA over longer periods leads to underestimation of time spent in the lower (SED) and the higher (VPA, MVPA) end of the intensity spectrum as compared to summation over shorter periods [23, 41, 42]. This effect clearly suggests that a short epoch is needed to capture children’s time spent across the intensity spectrum correctly, as supported by studies showing that a short epoch can capture information that is lost when applying longer epoch length [23, 43]. To the best of our knowledge, the present study is the first to compare PA trajectories in childhood using two different epoch lengths. Our findings show not only differences in SED and PA levels across epoch lengths, but also different trajectories across epochs for SED and all PA intensities. The most pronounced differences were found for MPA and MVPA, where steeper increases and larger differences over time were found for a 60-s (increase of 25 and 33 min/day from age 3 to 7, respectively) than a 1-s (increase of 5 and 15 min/day from age 3 to 7, respectively) epoch length, expectedly resulting from children participating in less sporadic and more continuous PA patterns with older age. This is consistent with more favourable changes in 1-min bouts of MPA than for total MPA (15-s epoch) from age 4.6 to 10.6 in a large Australian sample [44], and may be related to increased participation in organized sport with older age [45]. Given that previous longitudinal studies including multiple timepoints have applied 15- [8, 10, 15] or 60-s [12, 13] epoch lengths, the ICAD [5, 14] applies a 60-s epoch and the meta-analysis by Farooq [7] included studies with various epoch lengths, different epoch lengths is clearly a candidate for inconsistency in findings across studies. Consistent with previous studies showing that a 1-s epoch can capture information of relevance for metabolic health that 10- or 60-s epochs cannot [23, 43], we suggest that using a 1-s epoch in children is appropriate to more accurately capture time spent across the PA intensity spectrum.

We found that tracking coefficients (ICCs) across the 5 timepoints ranged from 0.47 to 0.59 for the total day. These estimates are in line with a previous systematic review [46] and a recent Australian study with 3 follow-up timepoints over 6 years [44]. Thus, our findings add to the literature showing moderate stability of PA and SED from early to mid childhood, reinforcing the importance of efforts to establish favourable activity levels during the early years to support long-term healthy movement behaviours. Interestingly, the ICCs observed over 5–6 years of follow-up in the present study and the study by Downing et al. [44] are similar to ICCs previously shown over multiple timepoints within a year in children [29, 47, 48]. This similarity of ICCs over the short and long term, raise the question what should be considered measurement error and actual behaviour change in assessment and understanding of habitual PA levels. If variation is similar over weeks or months (which principally could be considered measurement error) and years (which principally could be considered actual behaviour change), it could be argued that stability in PA over time is higher than previously believed, given that much of the variability over the long term could be considered random fluctuations and thus measurement error. The somewhat lower stability for weekdays, weekend days, preschool/school hours, and after school hours as compared to PA over the total day, probably reflect shorter monitoring periods (hours/day and/or days/week) and thus more variability. Particularly for weekend days, for which the lowest ICCs were found, this finding is expected and consistent with a previous study in children [49], given the inclusion of only 1–4 days of monitoring for each timepoint.

### Strengths and limitations

The main strength of this study was the inclusion of a relatively large sample of children who was followed annually for 5 years to establish PA trajectories for children aged 3 to 9 years. The study was specifically designed to capture the transition from preschool to school, having multiple timepoints of assessment both prior to and after the start of school to provide detailed evidence of shifts in activity levels across various settings. Children wore accelerometers for 14 consecutive days during the same period each year (autumn) to minimize variability in assessments, though such an extended monitoring period (i.e., ≥ 7 days) may be of minor importance for reliability of accelerometer-determined PA [29].

Though we invited all children from the included preschools in 3 municipalities that participated in PRESPAS [16] and the response rate was high, our findings are limited by not being a representative sample of children from a larger geographical area and with a more diverse sociodemographic background. We also found a higher parental education level in the follow-up sample compared to the larger PRESPAS sample. The lack of statistical significance testing of changes in PA and SED with age (i.e., 6 versus 5 years, 5 versus 4 years, etc.) might be considered a limitation, since we were not able to conclude with respect to whether differences between specific age groups were statistically significant. However, we did not put forward any hypotheses about specific changes between age groups, leaving such testing less meaningful. Thus, we have described trajectories and tested overall trends with age, including possible interactions between age and sex, type of day, time of day, and epoch length. We used a waking accelerometer monitoring protocol. Wear time was restricted to the hours between 06:00 and 23:59 to remove invalid wear time if children wore the accelerometer overnight. Given the age of the children was from 3 to 9 years, we believe that this restriction is appropriate to balance the risk of including invalid wear time if wearing the accelerometer during the night and the risk of missing wear time during the daytime. Still, this removal may have underestimated PA and/or SED for some children if they went to bed very late or got up very early in the morning. In general, little agreement exists on the most approrpiate data reduction protocols of accelerometry data in children (21) and alternative choices, beyond the influence of epoch length as shown herein, could affect our findings. PA may also be underestimated because accelerometers are not able to correctly capture activities like bicycling, swimming, and movements involving the upper body.

## Conclusion

In Norwegian children aged 3 to 9 years, total PA peaked at age 5, whereas intensity-specific PA peaked at ages 3 to 8 depending on sex, intensity, and, to some extent, epoch. Boys generally had more favourable PA levels and trajectories than girls, including a steeper increase and a later peak (7 years in girls and 8 years in boys) of moderate and vigorous intensities. SED increased from age 3 in girls and age 4 in boys. While trajectories were rather similar across weekdays and weekend days, they differed for preschool/school hours and after school hours and were also clearly affected by epoch. Our findings on PA trajectories and their stability suggest increased efforts should be devoted to establishing optimal conditions for PA at a young age. Finding ways to prevent decline in PA during the transition from preschool to primary school is important, especially for girls. Standardising epoch length will help when comparing PA and SED across studies.

## Supplementary Information

**Additional file 1.**

## Data Availability

The datasets used in the current study are available from the corresponding author on reasonable request.
