# Supplementary material for: Trajectories of physical activity and sedentary time in Norwegian children aged 3–9 years: a 5-year longitudinal study
Source: Int J Behav Nutr Phys Act. 2022 Jun 11;19:67. doi: 10.1186/s12966-022-01286-0 (PMC9188121; doi:10.1186/s12966-022-01286-0)
Supplement: Supplementary file 1 — Additional file 1. [file 12966_2022_1286_MOESM1_ESM.pdf]

Supplemental Table 1. Mean (SD) PA levels (percentage of wear time) for the total day across age and sex using a 1-second epoch length.

	Age						
	**3**	**4**	**5**	**6**	**7**	**8**	**9**
	**Boys**						
Wear days (n)	12.9 (1.5)	12.7 (1.8)	12.3 (2.1)	12.6 (3.0)	12.4 (2.8)	12.1 (2.6)	12.4 (2.1)
Wear time (min/day)	688 (39)	695 (32)	709 (38)	727 (51)	729 (45)	741 (41)	761 (41)
Total PA (cpm)	641 (99)	743 (162)	774 (182)	754 (182)	742 (167)	720 (188)	656 (172)
SED (%)	68.4 (3.4)	67.4 (3.9)	67.6 (4.2)	68.9 (4.2)	70.1 (3.7)	71.7 (4.2)	74.0 (4.1)
LPA (%)	22.3 (2.4)	21.9 (2.4)	21.1 (2.4)	20.0 (2.5)	18.8 (2.1)	17.5 (2.4)	16.1 (2.0)
MPA (%)	5.0 (0.9)	5.5 (0.9)	5.7 (1.0)	5.6 (1.0)	5.6 (1.1)	5.3 (1.1)	5.0 (1.2)
VPA (%)	4.2 (0.9)	5.1 (1.3)	5.6 (1.6)	5.5 (1.6)	5.5 (1.8)	5.5 (1.8)	4.9 (1.5)
MVPA (%)	9.3 (1.6)	10.7 (2.1)	11.3 (2.4)	11.1 (2.4)	11.1 (2.4)	10.8 (2.7)	9.9 (2.5)
	**Girls**						
Wear days (n)	12.7 (1.7)	12.8 (1.9)	12.6 (2.2)	12.3 (2.6)	12.6 (3.0)	11.8 (2.4)	12.0 (2.3)
Wear time (min/day)	665 (49)	696 (39)	701 (36)	708 (38)	721 (38)	732 (40)	751 (50)
Total PA (cpm)	625 (141)	693 (150)	720 (146)	695 (153)	704 (156)	633 (150)	603 (104)
SED (%)	69.9 (4.1)	69.4 (3.7)	70.0 (3.6)	71.1 (3.8)	72.0 (3.5)	74.0 (3.8)	75.3 (3.2)
LPA (%)	21.3 (2.9)	20.7 (2.1)	19.9 (2.3)	18.9 (2.3)	18.0 (2.0)	16.8 (2.4)	15.8 (2.1)
MPA (%)	4.7 (0.8)	5.1 (0.8)	5.0 (0.7)	5.0 (0.9)	4.9 (0.9)	4.6 (0.9)	4.6 (0.9)
VPA (%)	4.1 (1.1)	4.8 (1.3)	5.1 (1.2)	5.0 (1.3)	5.1 (1.3)	4.5 (1.2)	4.3 (0.9)
MVPA (%)	8.8 (1.8)	9.9 (2.0)	10.1 (1.8)	10.0 (2.1)	10.0 (2.0)	9.2 (1.9)	8.9 (1.6)

PA = physical activity; SED = sedentary time; LPA = light physical activity; MPA = moderate physical activity; VPA = vigorous physical activity; MVPA = moderate- to vigorous physical activity

Supplemental Table 2. Mean (SD) PA levels for weekdays across age and sex using a 1-second epoch length.

	Age						
	**3**	**4**	**5**	**6**	**7**	**8**	**9**
	**Boys**						
Wear days (n)	9.3 (0.9)	9.3 (1.2)	9.0 (1.5)	9.3 (2.1)	9.2 (2.1)	8.9 (1.8)	9.1 (1.5)
Wear time (min/day)	704 (46)	711 (36)	726 (40)	745 (49)	747 (43)	760 (42)	774 (42)
Total PA (cpm)	639 (99)	746 (171)	795 (192)	770 (193)	749 (156)	734 (181)	685 (178)
SED (min/day)	481 (40)	478 (37)	487 (43)	510 (52)	521 (43)	542 (48)	567 (48)
LPA (min/day)	158 (19)	157 (19)	155 (19)	150 (20)	142 (17)	134 (18)	127 (15)
MPA (min/day)	35 (7)	40 (7)	42 (8)	43 (8)	42 (8)	41 (8)	40 (10)
VPA (min/day)	30 (7)	37 (10)	42 (12)	42 (12)	41 (12)	42 (13)	40 (12)
MVPA (min/day)	65 (12)	76 (16)	83 (18)	84 (19)	84 (18)	84 (19)	80 (20)
	**Girls**						
Wear days (n)	9.5 (0.9)	9.3 (1.3)	9.2 (1.4)	9.2 (1.8)	9.3 (1.7)	8.7 (1.7)	8.8 (1.6)
Wear time (min/day)	675 (47)	712 (42)	718 (36)	729 (42)	741 (40)	755 (42)	776 (53)
Total PA (cpm)	628 (121)	704 (159)	732 (149)	705 (154)	717 (152)	653 (145)	622 (115)
SED (min/day)	471 (43)	492 (40)	501 (40)	516 (43)	531 (41)	556 (41)	582 (48)
LPA (min/day)	145 (22)	148 (18)	144 (18)	139 (18)	134 (16)	128 (21)	123 (20)
MPA (min/day)	32 (5)	36 (7)	36 (5)	37 (7)	37 (7)	36 (7)	36 (8)
VPA (min/day)	28 (7)	35 (10)	37 (9)	37 (10)	39 (10)	36 (9)	34 (7)
MVPA (min/day)	59 (11)	71 (16)	74 (13)	74 (15)	76 (15)	71 (15)	71 (13)

PA = physical activity; SED = sedentary time; LPA = light physical activity; MPA = moderate physical activity; VPA = vigorous physical activity; MVPA = moderate- to vigorous physical activity

Supplemental Table 3. Mean (SD) PA levels for weekend days across age and sex using a 1-second epoch length.

	Age						
	**3**	**4**	**5**	**6**	**7**	**8**	**9**
	**Boys**						
Wear days (n)	3.6 (1.1)	3.4 (1.0)	3.3 (1.2)	3.3 (1.3)	3.3 (1.1)	3.2 (1.2)	3.3 (1.0)
Wear time (min/day)	645 (48)	648 (50)	655 (68)	671 (81)	680 (75)	687 (77)	721 (74)
Total PA (cpm)	654 (152)	737 (188)	730 (212)	726 (235)	725 (260)	694 (268)	582 (223)
SED (min/day)	440 (50)	440 (48)	453 (62)	472 (79)	487 (78)	501 (77)	551 (75)
LPA (min/day)	144 (26)	141 (24)	133 (24)	129 (23)	122 (23)	116 (24)	109 (25)
MPA (min/day)	33 (7)	35 (8)	36 (10)	35 (9)	35 (11)	34 (10)	31 (10)
VPA (min/day)	28 (7)	32 (9)	34 (12)	35 (13)	36 (15)	36 (17)	31 (14)
MVPA (min/day)	61 (14)	67 (16)	70 (20)	70 (20)	71 (24)	70 (26)	61 (23)
	**Girls**						
Wear days (n)	3.1 (1.1)	3.4 (1.1)	3.4 (1.1)	3.2 (1.2)	3.3 (1.2)	3.1 (1.1)	3.2 (1.1)
Wear time (min/day)	631 (82)	651 (63)	652 (61)	646 (61)	666 (73)	667 (68)	675 (82)
Total PA (cpm)	638 (219)	670 (179)	694 (212)	667 (205)	663 (232)	572 (198)	564 (143)
SED (min/day)	445 (72)	456 (59)	464 (61)	466 (60)	486 (72)	503 (63)	513 (66)
LPA (min/day)	132 (29)	133 (20)	125 (22)	119 (22)	119 (22)	109 (21)	106 (23)
MPA (min/day)	29 (9)	32 (7)	31 (8)	31 (9)	30 (9)	28 (8)	29 (9)
VPA (min/day)	26 (11)	29 (10)	31 (10)	31 (11)	31 (12)	27 (10)	27 (9)
MVPA (min/day)	55 (19)	62 (16)	62 (17)	61 (19)	61 (19)	54 (17)	56 (18)

PA = physical activity; SED = sedentary time; LPA = light physical activity; MPA = moderate physical activity; VPA = vigorous physical activity; MVPA = moderate- to vigorous physical activity

Supplemental Table 4. Mean (SD) PA levels for preschool/school hours across age and sex using a 1-second epoch length.

	Age						
	**3**	**4**	**5**	**6**	**7**	**8**	**9**
	**Boys**						
Wear days (n)	9.4 (1.0)	9.5 (1.2)	9.2 (1.5)	9.6 (2.0)	9.4 (2.0)	8.9 (1.8)	9.1 (1.5)
Wear time (min/day)	402 (22)	412 (10)	413 (11)	412 (11)	411 (9)	411 (8)	409 (9)
Total PA (cpm)	676 (120)	820 (226)	882 (229)	841 (250)	824 (190)	804 (215)	745 (172)
SED (min/day)	266 (17)	263 (20)	262 (21)	269 (21)	275 (18)	282 (20)	288 (19)
LPA (min/day)	97 (15)	100 (14)	97 (12)	91 (14)	85 (10)	78 (12)	73 (10)
MPA (min/day)	22 (4)	26 (5)	27 (5)	27 (6)	26 (6)	25 (6)	25 (6)
VPA (min/day)	18 (4)	24 (8)	27 (9)	26 (9)	26 (8)	26 (9)	23 (7)
MVPA (min/day)	40 (8)	49 (12)	54 (13)	52 (14)	52 (13)	51 (13)	48 (12)
	**Girls**						
Wear days (n)	9.6 (0.9)	9.7 (1.2)	9.4 (1.4)	9.4 (1.8)	9.5 (2.2)	8.9 (1.8)	9.0 (1.6)
Wear time (min/day)	401 (20)	411 (13)	411 (13)	411 (10)	411 (9)	409 (10)	407 (8)
Total PA (cpm)	668 (131)	756 (191)	790 (177)	771 (191)	768 (175)	725 (187)	670 (147)
SED (min/day)	271 (19)	274 (19)	276 (18)	281 (19)	287 (16)	293 (15)	299 (17)
LPA (min/day)	92 (14)	92 (11)	89 (12)	84 (11)	78 (10)	73 (12)	67 (11)
MPA (min/day)	21 (4)	23 (4)	23 (4)	23 (5)	22 (5)	22 (5)	21 (5)
VPA (min/day)	18 (5)	22 (7)	24 (7)	23 (7)	24 (7)	22 (7)	20 (5)
MVPA (min/day)	38 (8)	45 (11)	46 (9)	47 (11)	46 (10)	44 (10)	41 (10)

PA = physical activity; SED = sedentary time; LPA = light physical activity; MPA = moderate physical activity; VPA = vigorous physical activity; MVPA = moderate- to vigorous physical activity

Supplemental Table 5. Mean (SD) PA levels for after school hours across age and sex using a 1-second epoch length.

	Age						
	**3**	**4**	**5**	**6**	**7**	**8**	**9**
	**Boys**						
Wear days (n)	8.5 (1.9)	8.4 (1.8)	8.4 (2.0)	8.9 (2.2)	8.9 (2.2)	8.8 (1.9)	9.1 (1.6)
Wear time (min/day)	248 (35)	250 (28)	264 (33)	274 (38)	283 (34)	295 (36)	315 (34)
Total PA (cpm)	629 (113)	688 (184)	719 (212)	721 (262)	693 (192)	686 (238)	654 (268)
SED (min/day)	174 (28)	177 (25)	187 (29)	197 (34)	206 (29)	220 (35)	240 (35)
LPA (min/day)	51 (10)	49 (9)	50 (9)	50 (10)	49 (9)	47 (9)	47 (8)
MPA (min/day)	12 (3)	12 (3)	13 (3)	13 (4)	14 (3)	14 (4)	14 (4)
VPA (min/day)	10 (3)	12 (4)	13 (4)	14 (6)	14 (5)	15 (6)	15 (7)
MVPA (min/day)	22 (6)	24 (6)	27 (7)	28 (9)	28 (8)	29 (9)	29 (10)
	**Girls**						
Wear days (n)	8.0 (1.5)	8.4 (1.9)	8.7 (1.7)	8.7 (2.0)	9.0 (2.3)	8.4 (1.7)	8.7 (1.7)
Wear time (min/day)	242 (30)	251 (30)	261 (29)	267 (30)	276 (34)	292 (34)	308 (44)
Total PA (cpm)	607 (154)	675 (194)	697 (214)	652 (190)	689 (233)	613 (200)	590 (171)
SED (min/day)	173 (28)	180 (27)	188 (26)	196 (26)	204 (31)	221 (32)	235 (37)
LPA (min/day)	49 (9)	48 (9)	48 (9)	47 (9)	47 (9)	47 (10)	47 (11)
MPA (min/day)	10 (2)	12 (3)	12 (3)	12 (3)	12 (3)	12 (3)	12 (3)
VPA (min/day)	10 (3)	12 (4)	13 (4)	12 (4)	14 (5)	13 (4)	13 (4)
MVPA (min/day)	20 (5)	23 (7)	24 (6)	24 (7)	26 (8)	25 (7)	25 (7)

PA = physical activity; SED = sedentary time; LPA = light physical activity; MPA = moderate physical activity; VPA = vigorous physical activity; MVPA = moderate- to vigorous physical activity

Supplemental Table 6. Mean (SD) PA levels for the total day across age and epoch length.

	Age						
	**3**	**4**	**5**	**6**	**7**	**8**	**9**
	**1-second epoch**						
Wear days (n)	12.8 (1.6)	12.7 (1.9)	12.5 (2.1)	12.4 (2.8)	12.5 (2.9)	11.9 (2.5)	12.2 (2.2)
Wear time (min/day)	678 (45)	695 (36)	705 (37)	718 (46)	725 (42)	737 (41)	756 (45)
Total PA (cpm)	634 (118)	718 (157)	748 (168)	724 (168)	723 (163)	677 (175)	632 (147)
SED (min/day)	468 (41)	476 (38)	485 (40)	503 (47)	516 (43)	537 (46)	564 (47)
LPA (min/day)	148 (20)	148 (17)	144 (18)	139 (18)	134 (16)	126 (19)	121 (17)
MPA (min/day)	33 (6)	37 (6)	38 (7)	38 (8)	38 (8)	37 (8)	36 (8)
VPA (min/day)	28 (7)	35 (9)	38 (10)	38 (11)	38 (11)	37 (12)	35 (10)
MVPA (min/day)	61 (12)	71 (15)	76 (16)	76 (17)	76 (17)	74 (18)	71 (16)
	**60-second epoch**						
Wear days (n)	12.8 (1.6)	12.7 (1.9)	12.5 (2.1)	12.4 (2.8)	12.5 (2.9)	11.9 (2.5)	12.2 (2.2)
Wear time (min/day)	680 (45)	697 (36)	707 (37)	719 (47)	728 (42)	739 (41)	759 (45)
Total PA (cpm)	631 (118)	715 (158)	745 (167)	721 (169)	721 (162)	674 (174)	629 (147)
SED (min/day)	227 (38)	225 (41)	235 (46)	264 (50)	285 (47)	315 (54)	350 (51)
LPA (min/day)	426 (43)	433 (40)	424 (40)	400 (44)	382 (39)	365 (47)	350 (45)
MPA (min/day)	22 (7)	31 (12)	37 (14)	44 (16)	47 (17)	45 (16)	46 (17)
VPA (min/day)	7 (4)	9 (6)	11 (7)	11 (8)	13 (8)	14 (10)	13 (8)
MVPA (min/day)	28 (10)	40 (16)	48 (19)	55 (22)	61 (23)	60 (24)	59 (23)

PA = physical activity; SED = sedentary time; LPA = light physical activity; MPA = moderate physical activity; VPA = vigorous physical activity; MVPA = moderate- to vigorous physical activity

Supplemental Table 7. Mean (SD) PA levels for the total day across age and sex using a 60-second epoch length.

	Age						
	3	4	5	6	7	8	9
	Boys						
Wear days (n)	12.9 (1.5)	12.7 (1.8)	12.3 (2.1)	12.6 (3.0)	12.4 (2.8)	12.1 (2.6)	12.4 (2.1)
Wear time (min/day)	690 (40)	697 (32)	711 (38)	729 (52)	731 (45)	743 (42)	763 (41)
Total PA (cpm)	638 (100)	740 (162)	771 (182)	751 (181)	739 (167)	718 (187)	654 (172)
SED (min/day)	226 (37)	221 (40)	229 (46)	263 (54)	284 (47)	310 (57)	353 (57)
LPA (min/day)	435 (41)	433 (43)	430 (42)	406 (45)	382 (40)	365 (44)	345 (41)
MPA (min/day)	23 (7)	34 (12)	42 (17)	49 (18)	52 (19)	52 (18)	51 (18)
VPA (min/day)	5 (4)	9 (6)	11 (7)	11 (9)	13 (9)	16 (13)	14 (9)
MVPA (min/day)	28 (9)	42 (16)	52 (22)	60 (24)	65 (26)	68 (28)	65 (26)
	Girls						
Wear days (n)	12.7 (1.7)	12.8 (1.9)	12.6 (2.2)	12.3 (2.6)	12.6 (3.0)	11.8 (2.4)	12.0 (2.3)
Wear time (min/day)	667 (49)	698 (40)	704 (36)	710 (41)	724 (38)	735 (41)	753 (50)
Total PA (cpm)	623 (140)	690 (150)	717 (146)	692 (152)	701 (156)	631 (149)	601 (104)
SED (min/day)	228 (39)	228 (41)	242 (46)	265 (46)	285 (47)	319 (50)	346 (43)
LPA (min/day)	413 (43)	433 (37)	419 (38)	395 (43)	383 (40)	365 (50)	355 (50)
MPA (min/day)	19 (7)	29 (12)	33 (10)	39 (13)	42 (13)	39 (11)	40 (12)
VPA (min/day)	8 (5)	9 (6)	11 (7)	10 (7)	13 (8)	12 (7)	12 (5)
MVPA (min/day)	27 (11)	38 (16)	43 (15)	50 (18)	55 (19)	51 (16)	52 (16)

PA = physical activity; SED = sedentary time; LPA = light physical activity; MPA = moderate physical activity; VPA = vigorous physical activity; MVPA = moderate- to vigorous physical activity

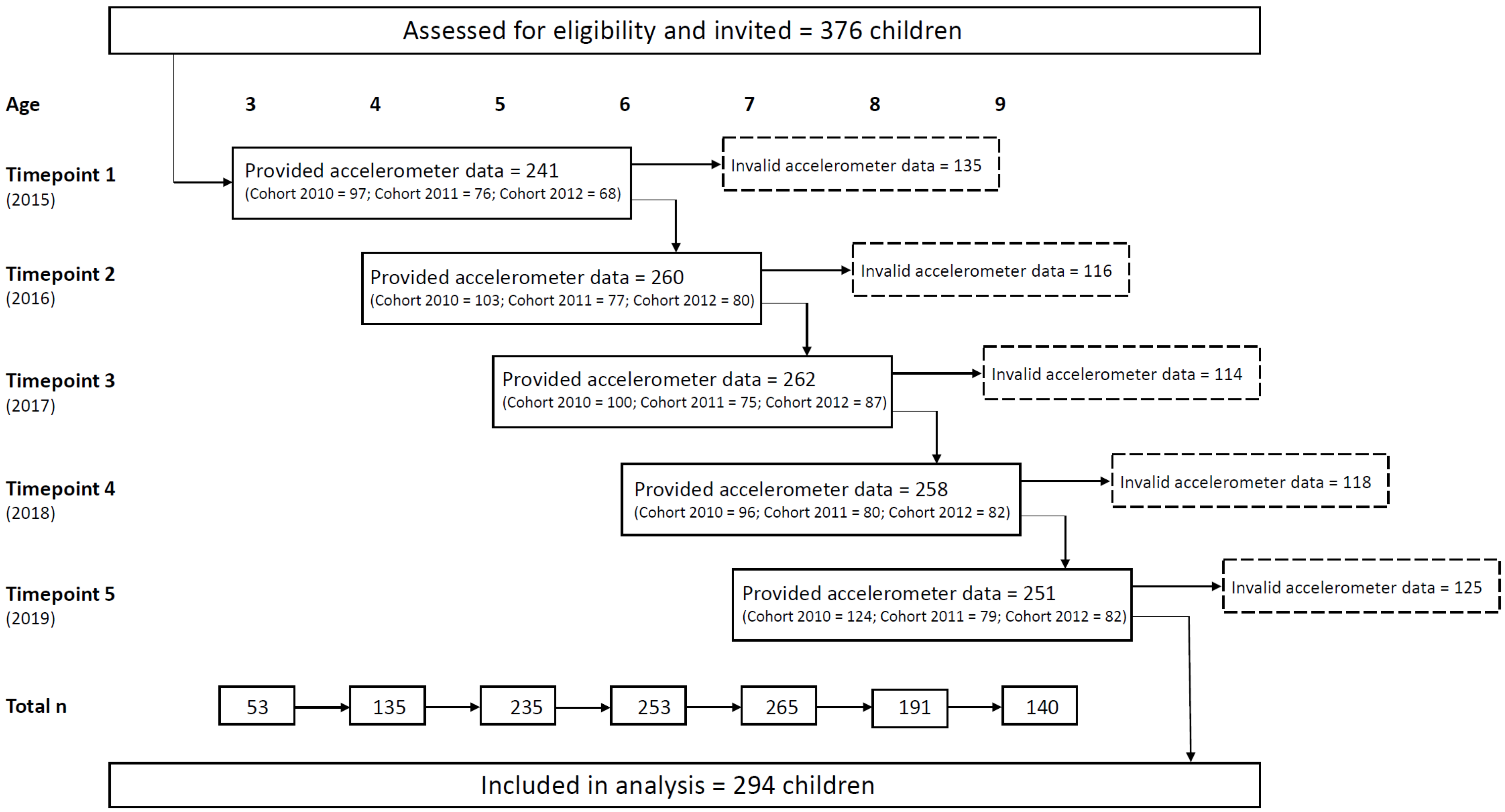

Supplemental Figure 1. Flow chart showing the number of children providing data across the follow-up period. 3 cohorts of children (born 2010, 2011, and 2012) provided data on 5 timepoints. Children providing valid data for all analysed contexts (≥ 3 weekdays, ≥ 1 weekend day, ≥ 3 weekday preschool/school hours, and ≥ 3 weekday after school hours) for at least 2 timepoints were included in the analysis.

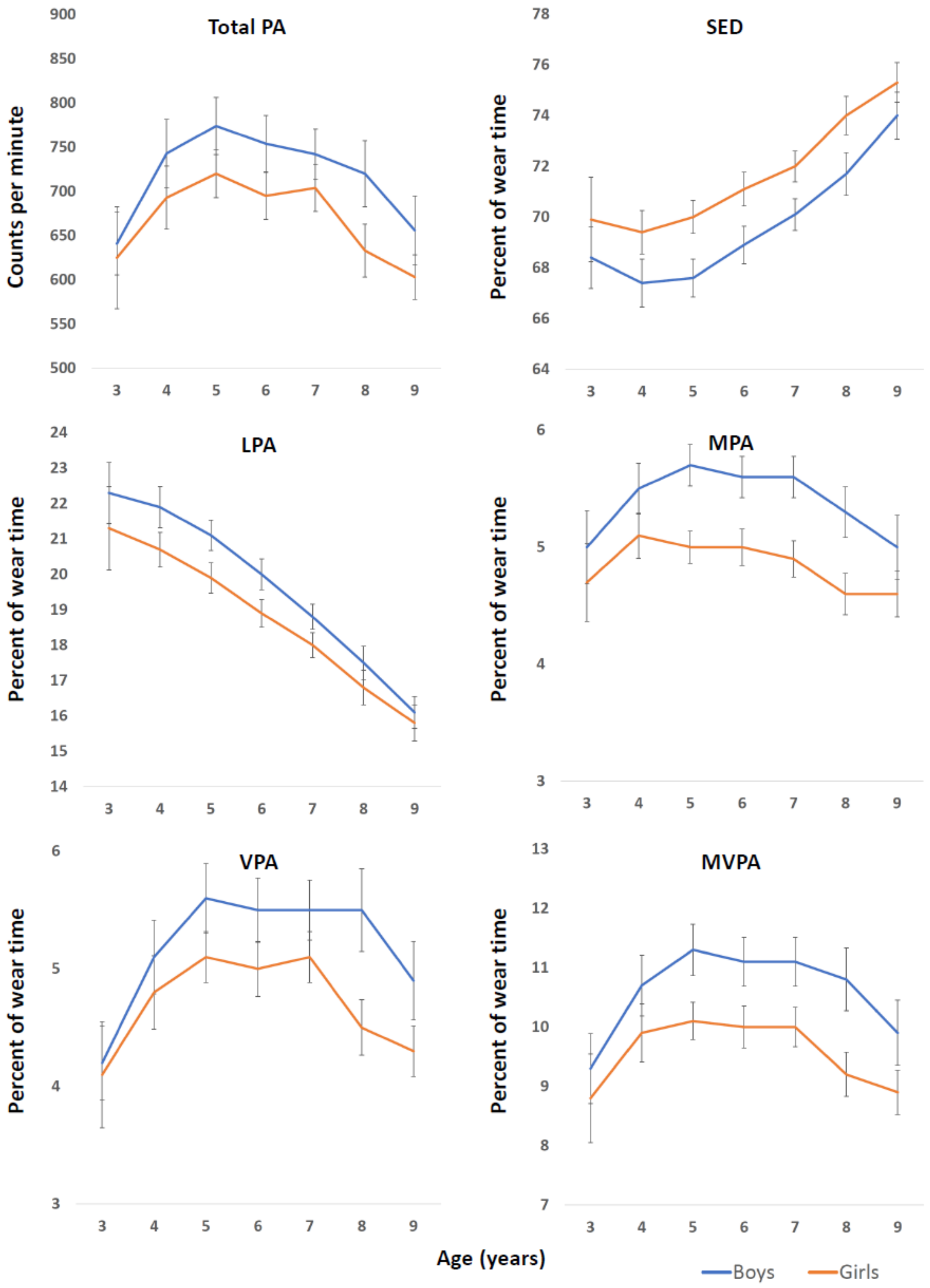

Supplemental Figure 2. Trajectories (means and 95% confidence intervals) for physical activity and sedentary time proportions (percent of valid wear time) in boys and girls aged 3–9 years using a 1-second epoch. PA = physical activity; SED = sedentary time; LPA = light physical activity; MPA = moderate physical activity; VPA = vigorous physical activity; MVPA = moderate- to vigorous physical activity

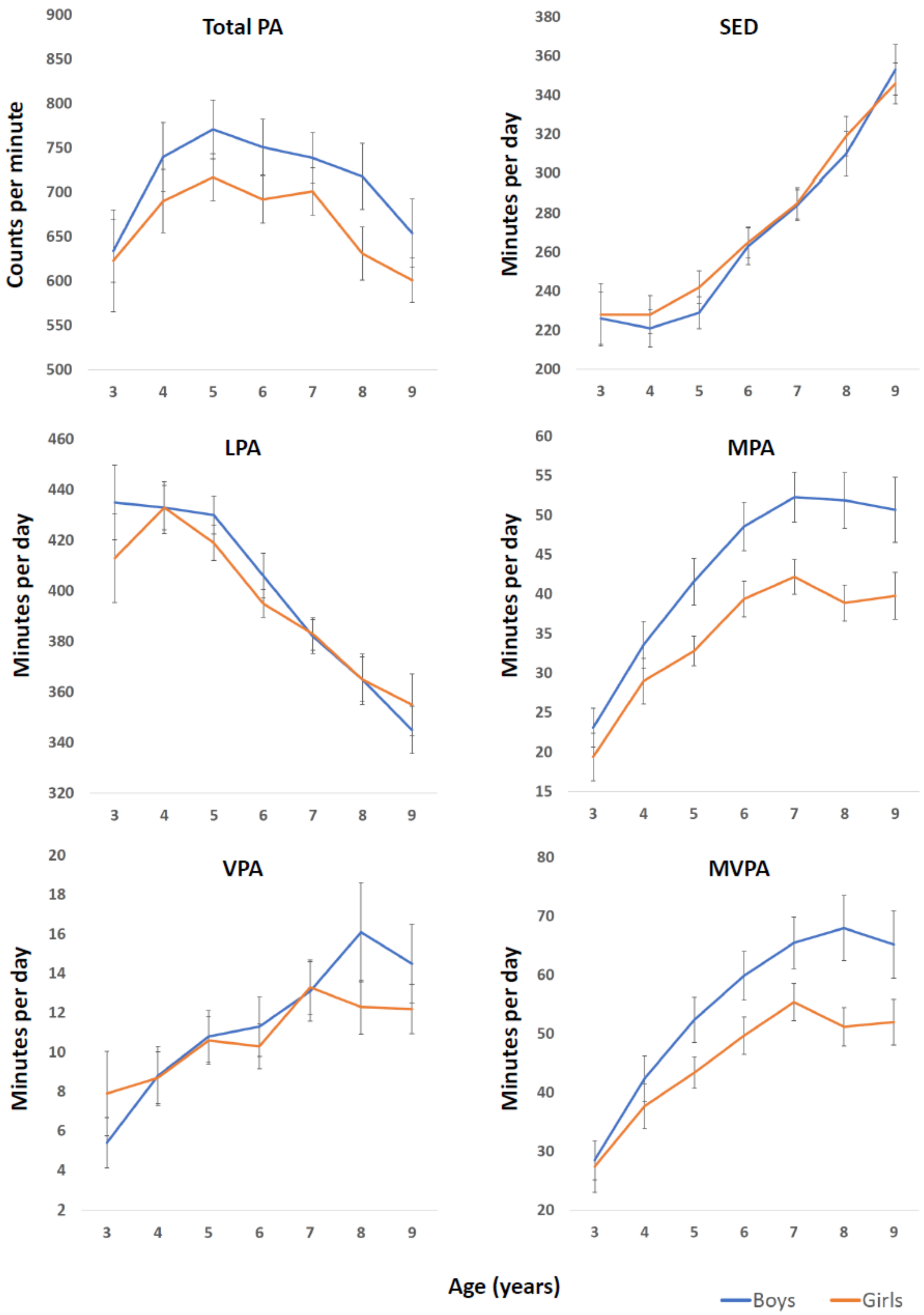

Supplemental Figure 3. Trajectories (means and 95% confidence intervals) for physical activity and sedentary time in boys and girls aged 3–9 years using a 60-second epoch. PA = physical activity; SED = sedentary time; LPA = light physical activity; MPA = moderate physical activity; VPA = vigorous physical activity; MVPA = moderate- to vigorous physical activity
